# Postdispersal nepotism in male long‐tailed macaques (*Macaca fascicularis*)

**DOI:** 10.1002/ece3.1839

**Published:** 2015-12-08

**Authors:** Livia Gerber, Michael Krützen, Jan R. de Ruiter, Carel P. van Schaik, Maria A. van Noordwijk

**Affiliations:** ^1^ Anthropological Institute & MuseumUniversity of ZurichWinterthurerstr 190CH‐8057ZurichSwitzerland; ^2^ Zen.nl, GroningenBeren 289714DVGroningenthe Netherlands

**Keywords:** Cooperation, kinship, long‐tailed macaques, *Macaca fascicularis*, nepotism

## Abstract

Cooperative behaviors are promoted by kin selection if the costs to the actor are smaller than the fitness benefits to the recipient, weighted by the coefficient of relatedness. In primates, cooperation occurs primarily among female dyads. Due to male dispersal before sexual maturity in many primate species, however, it is unknown whether there are sufficient opportunities for selective tolerance and occasional coalitionary support for kin selection to favor male nepotistic support. We studied the effect of the presence of male kin on correlates of male reproductive success (residence time, duration of high dominance rank) in non‐natal male long‐tailed macaques (*Macaca fascicularis*). We found that “related” (i.e., related at the half‐sibling level or higher) males in a group have a significantly higher probability to remain in the non‐natal group compared to males without relatives. Moreover, males stayed longer in a group when a relative was present at group entry or joined the same group within 3 months upon arrival. Males with co‐residing relatives also maintained a high rank for longer than those without. To our knowledge, this is the first demonstration of a potential nepotistic effect on residence and rank maintenance among non‐natal males in a social system without long‐term alliances.

## Introduction

Much animal cooperation is in the form of nepotism, in which individuals support relatives (kin). A behavior should be favored by kin selection if C < B*r*, where C is the fitness costs to the actor, B is the fitness benefits to the individual receiving the help, and *r* is the genetic relatedness between the two (Hamilton 1964a,b). The opportunities for nepotism therefore depend on opportunities for providing effective support and on the availability of relatives with high enough relatedness. Among vertebrates, nepotism is most prominent among cooperative breeders, with their relatively large numbers of full‐siblings (Cornwallis et al. 2010).

As in most mammals, long‐lasting supportive alliances in primates occur more often among females than among males (van Schaik 1996). This sex difference has traditionally been attributed to the fact that in many species, females are philopatric: They remain in their natal group for life. Indeed, their pervasive social alliances, with their positive fitness consequences (Silk et al. 2009, 2010), are generally built on kinship. Dispersing males, in contrast, often disperse before they become reproductively active (Lawson Handley and Perrin 2007), making it more difficult to maintain kin associations. In species with litters, fraternal littermates can co‐disperse and thus form lasting alliances (e.g. Caro 1990). Although among primates singleton births predominate, some species show natal co‐dispersal of alliance partners (Pope 1990; Schuelke et al. 2010; Perry 2012), most likely kin (Pope 1990; Perry 2012). Nonetheless, even where males do not form lasting alliances, we often see co‐dispersal, for instance, in Japanese monkeys (Kawanaka 1973), vervet monkeys (Cheney and Seyfarth 1983; J. Arsenau and E. Willems, unpubl.), and squirrel monkeys (Mitchell 1994), although it is clearly not universal (Chancellor et al. 2011). Moreover, individual dispersal into groups with maternal brothers has been reported for rhesus monkeys (Meikle and Vessey 1981), although it was not known whether this was selective.

There are, therefore, various species in which males do not form lasting alliances but where relatives nonetheless may end up in the same group after dispersal. Hence, the question arises whether in these species, there are enough opportunities for selective tolerance and occasional coalitionary support for kin selection to favor male nepotistic support.

This study focuses on Sumatran long‐tailed macaques (*Macaca fascicularis*), in which males disperse and form strict dominance hierarchies (van Noordwijk and van Schaik 1985). Paternities are highly skewed, with the top‐dominant male siring 60–100% of a group's offspring (de Ruiter et al. 1994; Engelhardt et al. 2006), making achieving the top rank and maintaining it for as long as possible the key determinant of male fitness. A young adult male singlehandedly (i.e. without coalition partners) can achieve top dominance by challenging the incumbent top‐dominant (van Noordwijk and van Schaik 1985). However, males who were already group members have a much higher success rate than males attempting to take over when joining a group. In both takeover situations, defensive coalitions are common and can be successful (van Schaik et al. 2006).

During natal dispersal, that is, the first dispersal event, which involves leaving their natal group, males of this species often accompany or follow peers (van Noordwijk and van Schaik 2001). Due to the high paternity concentration in this population, peers are often half‐siblings (de Ruiter et al. 1992) and dispersers can be highly related (de Ruiter and Geffen 1998). Males subsequently disperse again, often multiple times, but it is unknown whether these moves are kin‐biased. Dispersal allows males to move into the group where their mating success is maximized (van Noordwijk and van Schaik 2004), but might also enable renewed association with relatives. However, even if dispersal is not selectively aimed at joining male kin, this will often happen by default because males usually disperse into neighboring groups which are arranged along rivers like beads on a string (van Noordwijk and van Schaik 2001).

The aim of this study was therefore to use genetic analyses to examine the effect of the presence of male kin, defined as individuals related to the level of half‐siblings or more, in non‐natal long‐tailed macaque groups on important correlates of male reproductive success: residence length, and tenure of high dominance rank. The natal group and birth year were known for the majority of males, allowing us to identify peers and to test whether peers have a different effect on residence length and high‐rank tenure compared to relatives. Thereby, we were able to investigate whether kin recognition in male long‐tailed macaques may be based on phenotype matching or familiarity. However, pedigree information was available only for a subset of males. Thus, we needed to establish a method to estimate the relatedness of individual male dyads for whom parents were unknown.

## Material and Methods

### Determination of group membership

This study is based on behavioral and genetic data, collected in the Ketambe research area, northern Sumatra (3°1′N, 97°39′E, Gunung Leuser National Park, Aceh Tenggara, Indonesia). Demographic records and behavioral observations were collected on two well‐habituated groups of long‐tailed macaques (House and Antara) in a longitudinal study between 1976 and 1992 (van Noordwijk and van Schaik 1988, 1999, 2001). Both groups were followed for multiple days each month. During such 6–12 h follows, hourly sightings of all adults were recorded, as larger groups tend to fission into smaller foraging parties during the day, but mostly reunite toward the end of the day. Behavioral data were taken using focal animal sampling as well as scan sampling with 5‐min intervals (see e.g. van Schaik 1983; van Noordwijk and van Schaik 1985, 1988; Sterck and Steenbeek 1997). Births, dominance ranks (based on unidirectional bared‐teeth display of submission: de Waal 1977), and group membership of all individually known adults and immatures was assessed at least bi‐monthly from October 1976 to September 1977 and from December 1979 to the middle of 1992 during regular group‐ and individual follows. Males present in both 1977 and December 1979 were assumed to have been resident throughout this period. In order not to bias our analyses toward males with long tenure, we only included data on group membership and high‐rank tenure collected between January 1980 and February 1992.

In this population, males were never solitary for a prolonged period of time, most likely due to predation risk (van Schaik 1983; van Schaik et al. 1983) and because the promiscuous mating system provides all males at least some chances to mate. A male's group membership was considered to have ended when he was seen in another group or was absent during at least 2 months of group follows and not seen afterward (van Noordwijk and van Schaik 2001). Thus, male residence and the tenure of top‐dominant males (on average 25 months, van Noordwijk and van Schaik 2001) were known in detail, as well as the identity of the mothers of all individuals born in these groups since 1978 (van Noordwijk and van Schaik 1985, 2001).

In total, detailed behavioral data were collected on 24 individual males in the Antara group and 32 males in the House group. Group size and the number of non‐natal males varied within groups and over the study period: 9–35 individuals, including 1–10 non‐natal males for group Antara, and 30–54 individuals, including 5–14 non‐natal males for group House (van Noordwijk and van Schaik 1999). During the study period, we observed a total of 37 dispersal events out of groups House and Antara and 51 dispersals into one of our study groups. We observed 27 transfers between House and Antara and another 32 dispersal events into or out of these groups to or from nearby monitored groups (van Noordwijk and van Schaik 2001).

### Genetic sampling and laboratory procedures

Blood samples were taken from 94 individually known animals (31 females and 63 males, including juveniles) between 1984 and 1986 and in 1989. Due to the limited sampling period, we could not obtain genetic data on all the males we collected behavioral data for. Prior to blood withdrawal, the animals were trapped and anaesthetized (de Ruiter 1992). Some animals, mostly males who had left the two study groups before they were sampled, were anaesthetized using tele‐injection. Samples were stored at −80°C after arrival in the laboratory. Immediately after the samples were taken, they were centrifuged and stored in liquid nitrogen. Transport was by plane, still in liquid nitrogen.

We extracted DNA using Qiagen's DNeasy kit following the manufacturer's protocol with the following modifications. First, prior to all steps, we lysed red blood cells with Qiagen RBC lysis buffer (blood to buffer ratio 3:1). After incubating the lysed cells for 5 min, we centrifuged the solution to pellet white blood cells, which was followed by aspirating off the supernatant. Second, we incubated the re‐suspended white blood cell pellet overnight instead of the recommended 10 min.

We genotyped all individuals for which we were able to obtain blood samples for 19 autosomal microsatellite markers (Kikuchi et al. 2007; Higashino et al. 2009), using the polymerase chain reaction (PCR). PCRs were carried out in four multiplex reactions (Table S1). All PCR conditions are given in the Supporting Information. All autosomal microsatellite loci were checked for departure from Hardy–Weinberg equilibrium, linkage disequilibrium, and null alleles using Genepop 4.0. (Rousset 2008) (Supporting Information). To account for multiple tests, we applied a Bonferroni correction to test for linkage disequilibrium and for deviation from Hardy–Weinberg equilibrium (Rice 1989). Relatedness and paternity analyses are based on allele proportions of all 94 genotyped individuals.

All statistical analyses were carried out using a subset of individuals for which behavioral and genetic data were available (15 males in group Antara and 19 males in group House, consisting of 31 individuals as some males were observed in both groups).

### Generation of dyadic relatedness estimates

During the tenure of a top‐dominant male, many paternal half‐siblings will be sired within each group (de Ruiter et al. 1992), leading to a large cohort of offspring that are related at a half‐sibling level, that is, expected relatedness value of *r* = 0.25. Because this level of relatedness is sufficient to cause an effect on female fitness in cercopithecine primates (e.g. Silk et al. 2009), the same effect could therefore potentially hold for males. Hence, for the purpose of this paper, we defined non‐natal male relatives as individuals related at the level of half‐siblings or higher, and will henceforth refer to them as “related males”. According to this definition, however, “unrelated” dyads may also include distant kin.

In natural animal populations, dyads can be assigned to relatedness categories through a variety of relatedness estimators (Lynch 1988; Queller and Goodnight 1989; Ritland 1996; Lynch and Ritland 1999; Wang 2002, 2007). However, it has been shown that estimator performance (accuracy and precision) is markedly higher in populations with high variance in relatedness (Csillery et al. 2006). In order to test for potential nepotistic effects on the level of the dyad, we needed to maximize the ability to correctly assign individual dyads to the appropriate relatedness category. We therefore first carried out an analysis in kininfor, version 1 (Wang 2006), to determine the effect of the number of loci used on the power of relationship analysis (PW_*R*_). We estimated PW_*R*_ using the simulation approach for half‐siblings as primary hypothesis and unrelated individuals as null hypothesis, as well as for parent–offspring relationships and unrelated individuals, respectively. We simulated 1,000,000 genotypes, based on our allele frequencies and the genotyping errors for each marker, and set the confidence level to 0.05. Based on our final set of 18 microsatellite loci, the multilocus PW_*R*_ to discriminate between half‐siblings and unrelated individuals, and between parent–offspring pairs and unrelated individuals was 0.78 and 1.00, respectively. Given that our power to discriminate half‐siblings from unrelated dyads is not perfect, we used two approaches to categorize male dyads as “related” or “unrelated”.

We identified the best performing relatedness estimator for our dataset using the software coancestry, version 2.0 (Wang 2011). For this, we simulated for each relatedness estimator 1000 pairwise relatedness values (*r*‐values) for unrelated dyads (expected *r* (*r*_e_) = 0), half‐siblings (*r*_e_ = 0.25), full‐siblings (*r*_e_ = 0.5), and parent–offspring (*r*_e_ = 0.5). Allele frequencies for simulations were obtained from all 94 genotyped individuals. Simulated *r*‐values computed by the DyadML estimator showed small variance (*σ* ^2^ = 0.043) and a high correlation with the theoretically expected values (*R *=* *0.898) (Table S4). Thus, all subsequent analyses are based on the DyadML estimator.

### Assignment of males to “related” or “unrelated” category

We generated DyadML *r*‐values for the 31 males for which we had genetic and behavioral data. Males were assigned to the relationship categories “related” (i.e. half‐siblings or higher) or “unrelated” based on the range of *r*‐values of known (A1) and simulated (A2) dyadic genetic relatedness values of half‐sibling categories.

Our first approach (A1) is based on the range of observed pairwise genetic relatedness values of empirically determined half‐siblings. Maternal half‐siblings were identified from our long‐term demographic records and confirmed genetically in all cases where the mother's DNA was available (26 of 28 dyads, data not shown). Paternal half‐siblings were determined genetically through an independent paternity analysis using the software cervus 3.0 (Marshall et al. 1998; Kalinowski et al. 2007) (see Supporting Information and Table S5 for details). In total, we empirically identified 28 maternal and 51 paternal half‐sibling dyads involving a total of 38 individuals.

We then calculated dyadic *r‐*values for all identified half‐sibling dyads using the DyadML estimator (ranging from 0.00 to 0.49, mean = 0.26, SD = 0.012). In order to avoid the inclusion of incorrectly assigned males into subsequent analyses, we only classified male pairs ranging within the top 85 percentile (i.e. *r *≥* *0.14) of the distribution shown by the known half‐siblings as “related”. Males with an *r*‐value below 0.14 were classified as “unrelated”. We chose to set the cutoff at the 85 percentile because it is approximately the median value of our multilocus PW_*R*_ (0.78) and the correlation of the DyadML *r*‐values with the theoretically expected values (*R *=* *0.898). Using this approach A1, 24 males had at least one related male partner (*N* = 32, 19 of them were known half‐brothers from parentage analyses, mean *r*‐value = 0.30, SD = 0.11). The other 14 males did not have known related males according to our definition residing in the same group (mean *r*‐value = 0.02, SD = 0.03).

In our second approach (A2), we utilized the distribution of the 1000 simulated r‐values of unrelated and half‐siblings dyads each from the Coancestry analysis. We determined the lower 5 percentile of the distribution of half‐siblings (*r* ≤ 0.1843) as well as the upper 5 percentile of the unrelated dyads (*r* ≥ 0.0730). We then applied these cutoff values to our real dataset: Pairs of males with an *r*‐value above *r *=* *0.1843 were treated as “related”, whereas those with *r*‐values below *r *=* *0.0730 were regarded as “unrelated”. All male pairs with *r*‐values ranging between these values were excluded from further analyses. This approach identified 22 males with at least one relative and nine without a relative (*N* = 25, 18 of them were known half‐brothers from parentage analysis, mean *r*‐value = 0.36, SD = 0.10). To validate A2, we investigated how reliably this approach classifies the set of known half‐siblings. Of a total of 79 empirically determined pairs tested, 60 pairs were correctly assigned (75.95%), while only one pair was incorrectly identified (1.27%). Eighteen pairs (22.78%) fell between the cut‐off values and were thus not considered.

Statistical analyses for both approaches were highly consistent. Thus, results only from the first approach A1 are reported here. For results from A2, please refer to the SI.

### Comparison of residence time and high‐rank tenure of related and unrelated males

A mixed effects Cox model, computed in R 3.1.0 (Team R 2014) using *coxme* (Therneau 2015) showed that residence time in a group for males who dispersed from their natal group (median value of 53 months) was not different from the median value of 42 months for “non‐natal” (i.e. subsequent or secondary) dispersers (*χ* ^2^_ML_,: *P *=* *0.2; *N* = 19 natal dispersers, six censored; non‐natal dispersers: *N* = 18, 13 censored, individual ID and population as random effects, whereby ID was nested in population, Table 1). We therefore pooled natal and non‐natal dispersers in subsequent analyses to increase our sample size. We compared the residence time (in months) of males who had related males co‐residing for at least part of the time in the same group with the residence time of males without related males present in their group, using also a mixed effects Cox model (MECM). In this model, we entered the presence of relatives as a fixed effect and individual ID nested within population as random effects. The type of dispersal (i.e. natal or non‐natal), as well as the interaction between the presence of relatives and dispersal, was also added as a fixed effect to investigate whether the presence of relatives has a different effect on natal vs. non‐natal disperser. To assess whether related males or peers provided some sort of entry support for new immigrants, we tested whether males who joined or were joined by related males or peers at the time of immigration into a group (or within 3 months) were more likely to stay for one year than those without, using a general linear mixed effects model (GLMM) in R using *lme4* (Bates et al. 2014). This allowed us to enter individual ID and population (ID nested in population) as random effects. We calculated *P*‐values by performing maximum likelihood ratio tests of the full model including the binomial predictor variable (relatives/peers present and staying for a year) as response variable against a null model without the predictor variable. We tested both the effect of peers (defined as males born in the same group with a maximum age difference of two years) and relatives in order to investigate whether potential postdispersal nepotism is based on familiarity or phenotype matching. If only peers have an effect, this points toward familiarity as males differing in age might not know each other from their natal group because the older male might already have dispersed. However, if related males have an effect, kin recognition might be based on other mechanisms, such as phenotype matching. Males sometimes check out various groups before settling—following and associating with group members in the periphery of a group for some weeks and then moving on to another group (van Noordwijk and van Schaik 2001). Thus, we used the 3 months period as well as the point of entry to test for entry support by relatives.

**Table 1:** Results of the mixed effects Cox models

	*β*	SE	*z*‐value	*P*‐value
Residence time natal vs. non‐natal dispersers	−0.92	0.72	−1.27	0.2
Presence of relatives (yes/no)	−2.36	1.00	−2.36	0.018
Type of dispersal (natal/non‐natal)	−1.35	1.62	−0.84	0.40
Interaction between presence of relatives and type of dispersal	−0.93	1.83	0.51	0.61

We also investigated whether high‐ranking males co‐residing with related males could maintain a high rank for longer compared to ones without related males. For this, we created a dataset using only males who at some stage during their entire residence in a group achieved high rank (1–3), as this was a good predictor for paternity success (confirming earlier analyses using different techniques by de Ruiter et al. 1992; see also Supporting Information and Fig. S1). If high ranks were obtained during periods when less than five non‐natal males were residing in a group, only males from ranks 1 to 2 were used. This was performed because reproductive skew decreases with group size. In larger groups, the alpha male cannot monopolize all females, whereas this can be the case in smaller groups (de Ruiter 1992). We then compared the duration of the high‐rank tenure of males with at least one related partner in the group to those without, using a linear mixed effects model (LMM) in R using *lme4*. We entered tenure as fixed effect and individual ID nested in population as random effect. This analysis was carried out including males ranked 1–3 and also including the top two ranking males only (see Supporting Information). As with the GLMM, we obtained *P*‐values by comparing the null model without the predictor variable “relatives” with our full model in a maximum likelihood ratio test (refer to the Supporting Information for all R codes).

## Results

### Comparison of residence time of related and unrelated males

**Figure 1:**
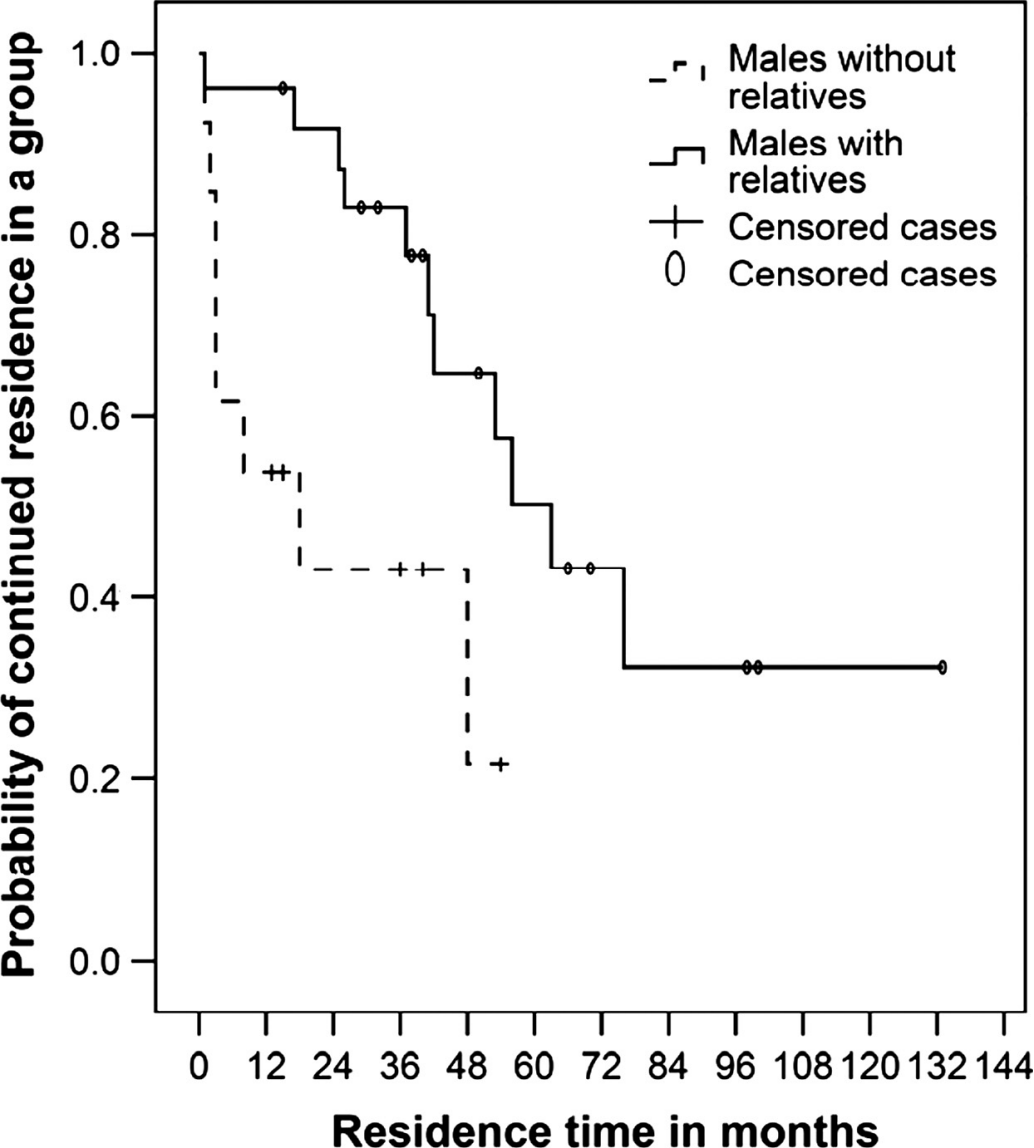
Probability of continued residence of adult males subsequent to entering a new group. The solid line and dashed line indicate the probabilities of non‐natal males with (*N* = 24) and without related males (*N* = 14), respectively, to stay in a new group.

Males with a related co‐residing partner in a group (N = 25; 14 censored) had a significantly higher probability to remain in the group compared to males without related partners (N = 12; 5 censored) (MECM, *χ* ^2^_ML_: *P *=* *0.018, Fig. 1, Table 1). On average, related male dyads co‐resided for 81.5% of their residence time (range 15–100%, *N* = 25). This result is not driven by natal dispersers, who have been shown to disperse into neighboring groups with peers (van Noordwijk and van Schaik 2001), as neither the type of dispersal (i.e. natal or non‐natal), nor the interaction between the presence of relatives and dispersal type had a significant effect on residence time (MECM, *χ* ^2^_ML Mode of Dispersal_: *P *=* *0.40, *χ* ^2^_ML Interaction_: *P *=* *0.61, Fig. 1, Table 1).

The difference in probability of continued residence in a group between related males and males without related partners is greatest within the first year (Fig. 1). Therefore, we tested whether the presence of related males at the moment of group entry affected whether males stayed for at least one year. We found that males appeared to be more likely to stay if a related male was present at group entry compared to males who did not have a male relative upon entry into a group (GLMM, *N* = 18 without related males, 66% stayed for a year, *N* = 17 with related males, 94% stayed for a year, *χ* ^2^_ML_: *P *=* *0.060, Table 2), although this was marginally nonsignificant. All males who had a relative at the time of group entry had a related male present throughout the first year in that group. We also tested whether a male entering a group without related males already present, but who was joined, within three months of group entry, by a related male, was more likely to reside for at least one year in that particular group. Here, we found a significant effect of co‐residence of related males (GLMM, *N* = 16 without related males, 54% stayed for a year, *N* = 19 with related males, 96% stayed for a year, *χ* ^2^_ML_: *P *=* *0.022, Table 2).

**Table 2:** Males joining a group where one or more relatives are already residing tend to stay longer compared to relatives. This effect is not observed when a male joins a group where one or more peers are present. Relative at entry: *χ* ^2^_ML_ = 3.52; relative within three months: *χ* ^2^_ML_ = 5.23; peer at entry: *χ* ^2^_ML_ = 0.20

	*β*	SE	*z*‐value	Pr (>¦*z*¦)	*P*‐value
Intercept	0.703	0.71	1.03	0.301	
Relative at entry yes/no	2.023	1.99	1.69	0.092	0.060
Intercept	0.661	1.10	0.60	0.548	
Relative within the first three months yes/no	2.807	2.26	1.24	0.214	0.022
Intercept	0.918	1.23	0.75	0.456	
Peers at entry yes/no	0.622	1.35	0.46	0.645	0.658

### Influence of peers on residence time

In an attempt to distinguish between the effect of relatedness and of familiarity, we also tested whether peers had an influence on residence time in the first 12 months of a male's residence in a group. Average residence time of males entering a group with a peer (including both natal and secondary disperses) was 10.7 months vs. 9.0 months for those entering a group without a peer. In contrast to the positive effect of related males on staying for more than a year, the decision to settle in a group seemed not to be strongly influenced by the presence of peers (note that this may include relatives: GLMM, *N* = 8 without peer, 50% stayed for a year, *N* = 16 with peer, 89% stayed for a year, *χ* ^2^_ML_: *P *=* *0.658, Table 2).

### Influence of co‐residing related males on high‐rank tenure

**Figure 2:**
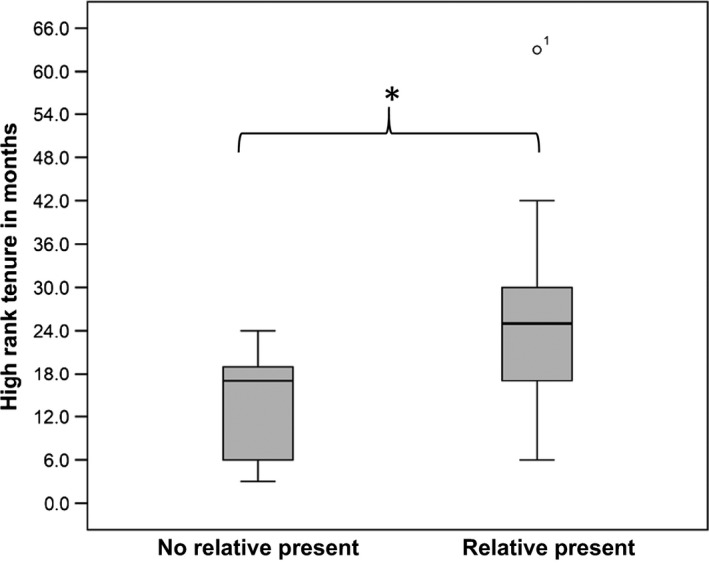
Effects of related males present in a group on high‐rank tenure. High‐ranking males (rank 1–3) with related males in the same group maintain a high rank significantly (P = 0.018) longer compared to males without related males.

Males co‐residing with one (*N* = 8) or two related males (*N* = 2) maintained a high rank (including ranks 1–3) for longer compared to males without related males (LMM, *N* = 7 without related males, *N* = 10 with related males, Antara and House group combined, *χ* ^2^_ML_: *P *=* *0.029, Fig. 2; Table 3 see Supporting Information for ranks 1 and 2 only). In this sample, related males were present on average for 81% (range 17–100%, *N* = 7) of a male's tenure.

**Table 3:** The presence of relatives has a significant effect on high‐rank tenure: *χ* ^2^_ML_ = 4.78

	*β*	SE	*t*‐value	*P*‐value
Intercept	14.2	4.08	3.48	
Predictor variable (Relative Yes/No)	12.7	5.76	2.20	0.029

## Discussion

Male nepotism in mammals should be affected by the opportunity for associations among relatives and the importance of coalitions for mating access to females. Where males disperse, it is harder for males to maintain associations, unless the species is polytokous (giving birth to multiple young at a time) and littermates can disperse as a cohort. Indeed, as noted in the introduction, the best evidence for potential postdispersal nepotism comes from polytokous species with male alliances. There is also evidence from monotokous (single young at a time) group‐living animals with high paternity concentration, where paternal half‐siblings are numerous and have the opportunity to co‐disperse and form long‐term alliances, moving from group to group (Pope 1990; van Belle et al. 2012).

However, so far, there has been no evidence for postdispersal nepotistic effects in a situation where females give birth to singletons, males disperse mostly independently, and male alliances are not essential for fitness. In long‐tailed macaques, females have single offspring, and males disperse independently and multiple times during their life, and do not form long‐lasting alliances, unlike some other macaques (e.g. Schuelke et al. 2010). In this study, we therefore examined two correlates of fitness, residence time and high‐rank tenure, in this species. We found potential evidence for potential postdispersal nepotism in males dispersing alone, including 19 cases where both males had transferred at least once more after their initial emigration from the natal group. Males with related males in a non‐natal group (half‐sibling level or higher) retained high‐rank positions for longer and stayed in the group for longer before dispersing again. Holding high rank for longer increases expected reproductive success because top dominants keep siring most offspring, independent of how long a male has occupied the rank position (J. R. de Ruiter, unpubl.). Even if a male cannot be highest ranking, he still might benefit from residing in a group, where he can improve his reproductive success by awaiting future opportunities where a higher rank is achievable, protecting his own offspring (van Noordwijk and van Schaik 1988), enhancing the success of relatives, or getting the odd chance to sire (see Fig. S1). Thus, the more limited potential for nepotism in males did not prevent males from somehow supporting their kin when this was possible. This result is, to our knowledge, the first demonstration of potential effective postdispersal nepotism among males in a social system where males do not form long‐term alliances.

It could be argued that we have the causation backwards, and that when relatives join at a certain constant probability, those residence times that are longer are more likely to be those in which a relative was also found at any time. This possibility is remote, however, because in most cases, the relatives were already present when the male entered the group. And where this was not the case, as in the analysis of tenure of high rank, the relatives were actually present most of the time, rather than for a small proportion, if they happened to be more likely to immigrate when a male has a longer tenure. Thus, our results are not an artifact of how we measured the effect of the presence of relatives.

The most detailed behavioral data on this population were largely collected in a different period than in the short period of collecting genetic data, so we were unable to confirm the nature of the kin support for the males in the sample. However, earlier data collected on our study groups showed that behavioral coalitions are especially found when top‐ranked males are threatened, a context in which we indeed recorded a clear kinship effect (van Noordwijk and van Schaik 2001). We also found a kinship effect for the early stage of immigration, which might be due to either passive tolerance or active support from other males. Earlier studies provided anecdotal evidence that recent immigrants receive agonistic protection from older familiar males, whereas peers initially maintain proximity, groom, and play with each other in their new group (van Noordwijk and van Schaik 2001). Clearly, future work is needed to substantiate the behavioral mechanisms of the kinship effects on tenure length and residence time documented here and whether male dispersal is selectively directed towards groups containing related males. Nonetheless, males could help kin in ways that do not necessarily compromise their fitness: refrain from attacking them when they immigrate and support them when they are high‐ranking and challenged (which, if successful, means that they themselves will also retain a higher rank).

Effective nepotism requires kin recognition. Maternal half‐siblings can easily estimate relatedness using association patterns (Langergraber 2012), provided they are not so far apart in age that the older one already left their natal group before the younger one was born. Recognition of paternal half‐siblings requires some form of phenotype matching, for which there is increasing evidence in nonhuman primates (Kessler et al. 2012; Widdig 2013; Pfefferle et al. 2014a,b), even though it stays controversial (Wikberg et al. 2014). Similarly, our results suggest that the kin effect on residence time is not simply due to familiarity, as the presence of peers at immigration did not significantly affect whether a male stayed in a group for at least a year, whereas the presence of related males did.

Having established the basic effect of postdispersal male nepotism, it would be interesting to examine its reach. Unfortunately, we did not have enough data to examine whether males also recognize half‐siblings they never met before in order to investigate the mode of kin recognition (i.e., phenotype matching or familiarity). Also missing is information on whether aging males support their sons when they meet again in a group, as this is known from father–offspring affiliations in the natal group of the offspring (Langos et al. 2013). To assess this, we need both experiments and studies with a large number of groups, and genetically identified well‐studied individuals with known pedigrees under long‐term behavioral observation.

## Conflict of Interest

None declared.

## Supporting information


**Table S1.** Distribution of the 19 autosomal microsatellite markers over the multiplexes.
**Table S2.** Genetic diversity indices for autosomal markers including all 94 individuals.
**Table S3.** Estimated proportions of null alleles per locus.
**Table S4.** Mean, Variance, and mean squared error (MSE) of simulated r‐values for the four relationship categories and over all relationship categories together.
**Table S5**. Input parameters for paternity assignments and critical Δ criteria for relaxed and strict paternity assignments calculated from cervus simulations.
**Table S6**. In the presence of a related male the two top‐ranking males can maintain their rank significantly longer compared to males without a related male: χ^2^_ML_ = 4.18.
**Table S7**. Results from the Mixed Effects Cox model of A2.
**Table S8**. Relative at entry: χ^2^_ML_ = 7.22.
**Figure S1.** Number of offspring sired per male rank: 100% of assigned offspring was sired by the top two ranking males in Antara group and 81% by the top three in House group.
**Figure S2.** Effects of related males present in a group on high‐rank tenure. High‐ranking males (rank 1 and 2) with related males in the same group maintain a high rank for longer compared to males without co‐residing related males.
