# Supplementary material for: Postdispersal nepotism in male long‐tailed macaques (Macaca fascicularis)
Source: Ecol Evol. 2015 Dec 8;6(1):46–55. doi: 10.1002/ece3.1839 (PMC4716510; doi:10.1002/ece3.1839)
Supplement: Supplementary file 1 — Table S1. Distribution of the 19 autosomal microsatellite markers over the multiplexes. Table S2. Genetic diversity indices for autosomal markers including all 94 individuals. Table S3. Estimated proportions of null alleles per locus. Table S4. Mean, Variance, and mean squared error (MSE) of simulated r‐values for the four relationship categories and over all relationship categories together. Table S5. Input parameters for paternity assignments and critical Δ criteria for relaxed and strict paternity assignments calculated from cervus simulations. Table S6. In the presence of a related male the two top‐ranking males can maintain their rank significantly longer compared to males without a related male: χ2 ML = 4.18. Table S7. Results from the Mixed Effects Cox model of A2. Table S8. Relative at entry: χ2 ML = 7.22. Figure S1. Number of offspring sired per male rank: 100% of assigned offspring was sired by the top two ranking males in Antara group and 81% by the top three in House group. Figure S2. Effects of related males present in a group on high‐rank tenure. High‐ranking males (rank 1 and 2) with related males in the same group maintain a high rank for longer compared to males without co‐residing related males. [file ECE3-6-046-s001.docx]

**Supporting Information**

**Polymerase Chain Reaction Multiplex Setup**

We developed our microsatellite multiplex polymerase chain reactions (PCR) based on 19 autosomal microsatellite markers described in Higashino (2009). A fluorescent dye (Applied Biosystems, FAM: blue, NED: yellow, VIC: green, PET: red) was attached to the 5’ end of forward primers. To enhance 3’ adenylation, we added ‘pig tails’ (Brownstein *et al.* 1996) (5’-GTTT-3’) to the 5’ end of reverse primers.

| **Multiplex reaction** | **FAM** | **NED** | **VIC** | **PET** |
| --- | --- | --- | --- | --- |
| MP1 | G09628 G07916 MFA0881 | G07956  MFA0825 | G08816 | G09003 |
| MP2 | G09378 MFA0651 | MFA0293 | MFA0305 MFA0908 | G08287 |
| MP3 | G08011  G09022 |  | G09598 | MFA0834 |
| MP4 | MFA0676 G08794 |  |  |  |

**Table S1:** Distribution of the 19 autosomal microsatellite markers over the multiplexes. FAM, NED, VIC, PET = ABI dye labels.

Polymerase chain reactions consisted of 1 µl template DNA, 0.25 µl primer mix (concentration of each primer 10 µM), 5 µl Multiplex Mastermix (Qiagen), and 3.75 µl ddH_2_O. All PCRs were carried out on a Veriti 96 well Thermal Cycler (Applied Biosystems) with the following PCR profile: initial denaturation at 95°C for 15 minutes, 35 amplification cycles at 94°C for 30 seconds, 58°C / (59°C for MP1) for 90 seconds, and 72°C for 1 minute. We carried out a final extension step for 30 minutes at 60°C. Each sample was diluted between 50 and 100 times with ddH_2_O, followed by adding 10 μl HiDi formamide and 0.07 μl GS500 LIZ size standard per sample. Samples were analysed on ABI 3730 Genetic Analyzer.

**Population Genetic Analyses**

Genetic diversity estimates were obtained using the software GenAlEx, 6.0 (Peakall & Smouse 2006) and Cervus 3.0. (Marshall *et al.* 1998) (Table S2). We checked all autosomal microsatellite loci for departure from Hardy-Weinberg equilibrium and the occurrence of linkage disequilibrium using Genepop 4.0. (Rousset 2008). To account for multiple tests, we applied a Bonferroni correction (Rice 1989). No linkage disequilibrum or deviation from Hardy-Weinberg equilibrium was detected in the 19 markers.

| **Locus** | **Repeat motif** | **Na** | **Ne** | **Typing success** | **Ho** | **He** | **PIC** | **AR** |
| --- | --- | --- | --- | --- | --- | --- | --- | --- |
| MFA0908 | Di | 12 | 6.929 | 97.8% | 0.851 | 0.857 | 0.836 | 11.747 |
| MFA0881 | Di | 9 | 5.430 | 79.6% | 0.806 | 0.820 | 0.790 | 9.000 |
| MFA0834 | Di | 12 | 3.596 | 97.8% | 0.667 | 0.720 | 0.698 | 11.775 |
| MFA0825 | Di | 9 | 3.875 | 97.8% | 0.747 | 0.745 | 0.702 | 8.619 |
| MFA0676 | Di | 7 | 3.249 | 97.8% | 0.747 | 0.694 | 0.653 | 6.815 |
| MFA0651 | Di | 12 | 6.781 | 96.8% | 0.859 | 0.851 | 0.829 | 11.500 |
| MFA0305 | Di | 9 | 2.700 | 98.9% | 0.625 | 0.647 | 0.617 | 8.926 |
| MFA0293 | Di | 8 | 3.564 | 95.7% | 0.553 | 0.710 | 0.669 | 7.942 |
| G09628 | Tetra | 10 | 5.199 | 97.8% | 0.897 | 0.813 | 0.792 | 10.000 |
| G09598 | Tetra | 7 | 3.887 | 96.8% | 0.782 | 0.751 | 0.707 | 6.814 |
| G09378 | Tetra | 9 | 4.786 | 100.0% | 0.798 | 0.802 | 0.775 | 8.992 |
| G09022 | Tetra | 9 | 3.764 | 88.2% | 0.475 | 0.728 | 0.688 | 8.885 |
| G09003 | Tetra | 6 | 3.554 | 98.9% | 0.716 | 0.717 | 0.661 | 5.806 |
| G08116 | Tetra | 8 | 2.615 | 97.8% | 0.506 | 0.627 | 0.595 | 7.803 |
| G08794 | Tetra | 11 | 7.033 | 97.8% | 0.908 | 0.862 | 0.841 | 10.995 |
| G08287 | Tetra | 9 | 5.938 | 100.0% | 0.809 | 0.834 | 0.809 | 8.798 |
| G08011 | Tetra | 7 | 3.318 | 93.5% | 0.631 | 0.704 | 0.642 | 6.828 |
| G07956 | Tetra | 12 | 6.934 | 88.2% | 0.888 | 0.860 | 0.839 | 11.895 |
| G07916 | Tetra | 8 | 5.537 | 96.8% | 0.849 | 0.824 | 0.796 | 7.999 |

**Table S2:** Genetic diversity indices for autosomal markers including all 94 individuals. Na = number of alleles, Ne= number of effective alleles, Ho = observed heterozygosity, He = expected heterozygosity, PIC = polymorphic information content, AR = allelic richness.

We then estimated the occurrence of null alleles using Genepop 4.0. (Rousset 2008) and by comparing genotypes of known mother-offspring pairs (N=34) as suggested by Dakin and Avise (2004) (Table S5). Marker G09022 had a high occurrence of null alleles using both methods and was thus excluded for all further analyses.

| **Locus** | **NA1** | **NA2** |
| --- | --- | --- |
| MFA0908 | 0 | 0.030 |
| MFA0881 | 0 | 0.056 |
| MFA0834 | 0 | 0.047 |
| MFA0825 | 0 | 0.037 |
| MFA0676 | 0 | 0.034 |
| MFA0651 | 0.0217 | 0.102 |
| MFA0305 | 0.0217 | 0.033 |
| MFA0293 | 0 | 0.178 |
| G09628 | 0 | 0.019 |
| G09598 | 0 | 0.078 |
| G09378 | 0 | 0.070 |
| G09022 | 0.0889 | 0.151 |
| G09003 | 0 | 0.002 |
| G08116 | 0.0215 | 0.082 |
| G08794 | 0.0215 | 0.013 |
| G08287 | 0 | 0.067 |
| G08011 | 0 | 0.055 |
| G07956 | 0 | 0.023 |
| G07916 | 0.0215 | 0.068 |

**Table S3:** Estimated proportions of null alleles per locus. NA1 = estimated by Mother-Offspring comparison, NA2 calculated by Genepop).

**Coancestry simulations to identify best-performing relatedness estimator**

Based on the simulations in Coancestry, we found that the DyadML and TrioML estimators generally had the smallest variances and highest accuracies (Table S4). The overall results do not differ regardless of whether DyadML or TrioML were used. In this paper, we only report results based on DyadML.

| N=1000 | **TrioML** | **Wang** | **Lynch&Li** | **L&R** | **Ritland** | **Q&G** | **DyadML** | **Expected Value UR** |
| --- | --- | --- | --- | --- | --- | --- | --- | --- |
| Mean | 0.032 | 0.003 | 0.005 | 0.003 | 0.002 | -0.007 | 0.042 | 0.000 |
| Variance | 0.002 | 0.016 | 0.017 | 0.007 | 0.008 | 0.015 | **0.004** |  |
| MSE | 0.003 | 0.016 | 0.017 | 0.007 | 0.008 | 0.015 | 0.005 |  |

| N=1000 | **TrioML** | **Wang** | **Lynch&Li** | **L&R** | **Ritland** | **Q&G** | **DyadML** | **Expected Value HS** |
| --- | --- | --- | --- | --- | --- | --- | --- | --- |
| Mean | 0.225 | 0.241 | 0.242 | 0.242 | 0.248 | 0.243 | 0.255 | 0.250 |
| Variance | 0.014 | 0.016 | 0.017 | 0.021 | 0.037 | 0.017 | **0.014** |  |
| MSE | 0.015 | 0.016 | 0.017 | 0.021 | 0.037 | 0.017 | 0.014 |  |
|  |  |  |  |  |  |  |  |  |
| N=1000 | **TrioML** | **Wang** | **Lynch&Li** | **L&R** | **Ritland** | **Q&G** | **DyadML** | **Expected Value FS** |
| Mean | 0.452 | 0.482 | 0.481 | 0.471 | 0.487 | 0.481 | 0.483 | 0.500 |
| Variance | 0.013 | 0.015 | 0.015 | 0.026 | 0.082 | 0.015 | **0.013** |  |
| MSE | 0.016 | 0.015 | 0.015 | 0.026 | 0.083 | 0.016 | 0.014 |  |

| N=1000 | **TrioML** | **Wang** | **Lynch&Li** | **L&R** | **Ritland** | **Q&G** | **DyadML** | **Expected Value PO** |
| --- | --- | --- | --- | --- | --- | --- | --- | --- |
| Mean | 0.495 | 0.484 | 0.483 | 0.475 | 0.497 | 0.484 | 0.507 | 0.500 |
| Variance | **0.002** | 0.005 | 0.007 | 0.018 | 0.064 | 0.006 | **0.002** |  |
| MSE | 0.002 | 0.005 | 0.007 | 0.018 | 0.064 | 0.006 | 0.002 |  |
|  |  |  |  |  |  |  |  |  |
| N=4000 | **TrioML** | **Wang** | **Lynch&Li** | **L&R** | **Ritland** | **Q&G** | **DyadML** | **Averaged expected value over all categories** |
| Mean | 0.301 | 0.301 | 0.301 | 0.297 | 0.305 | 0.301 | 0.322 | 0.313 |
| Variance | 0.042 | 0.054 | 0.055 | 0.056 | 0.088 | 0.054 | **0.043** | 0.043 |
| MSE | 0.009 | 0.013 | 0.014 | 0.019 | 0.047 | 0.014 | 0.009 |  |

**Table S4:** Mean, Variance, and mean squared error (MSE) of simulated r-values for the four relationship categories and over all relationship categories together. Smallest variances per relationship category are given in bold. Variances are smallest throughout for the TrioML and DyadML estimator. Estimators are TrioML (Wang 2007) and DyadML (Wang 2002), Lynch & Li (Lynch 1988), L&R = Lynch & Ritland (Lynch & Ritland 1999), Ritland (Ritland 1996), Q&G = Queller & Goodnight (Queller & Goodnight 1989). UR=unrelated, HS=half sibling, FS=full sibling, PO = parent/offspring.

**Paternity assignments**

Paternities were assigned using Cervus 3.0 (Marshall *et al.* 1998). To be conservative and to account for extra group paternities, we set the number of candidate fathers to 100. We had 56 males as candidate fathers, hence the proportion of candidate fathers sampled is 0.56. The proportion of mistyped loci was calculated from 83 microsatellite loci that were amplified independently twice. To consider for the large amount of relatedness among candidate fathers, we ran the analysis by assuming that half of the candidate males are related. We set the relatedness to the average r-value of known half-siblings (r=0.26). We tested 39 offspring and identified 28 paternities based on a 95% significance level. This discrepancy was expected because no genetic samples were available for some known high-ranking males. Table S8 summarises the Cervus input and critical Δ values calculated from the simulation.

| **Input Parameter** | **Value** |
| --- | --- |
| Number of offspring | 39 |
| Number of candidate fathers | 100 |
| Proportion of candidate fathers sampled | 0.56 |
| Proportion of loci typed | 0.96 |
| Proportion of loci mistyped | 0.01 |
| Minimum number of typed loci | 10 |
| Proportion of related candidate males | 0.5 |
| Relatedness | 0.26 |
| Critical Δ for 95% confidence assignment | 6.51 |
| Critical Δ if mother was sampled | 5.97 |
| Critical Δ for 85% confidence assignment | 2.53 |
| Critical Δ if mother was sampled | 1.16 |

**Table S5**: Input parameters for paternity assignments and critical Δ criteria for relaxed and strict paternity assignments calculated from Cervus simulations.

**Effect of rank on paternity success**

The effect of rank on paternity success was strong in both groups, with the top-dominant male siring the largest proportion of offspring, followed by the males holding ranks two (House and Antara groups) and three (House group) - see Fig S1 (*cf.* de Ruiter *et al*. 1994).

On average, 8.4 non-natal males were resident in the House group at the time of conception for the 16 offspring for which we could assign paternities. The ‘medium’ and ‘low’ category hence consisted of 2.7 males each. Males ranked lower than three sire a negligible number of offspring (Fig S1). Thus, we tested the influence of relatives on high rank tenure over the highest three and two ranks. Our sample size did not allow to test the influence of relatives on top-dominant male tenure (rank 1: N=6, rank 2: N=7, rank 3: N=7).


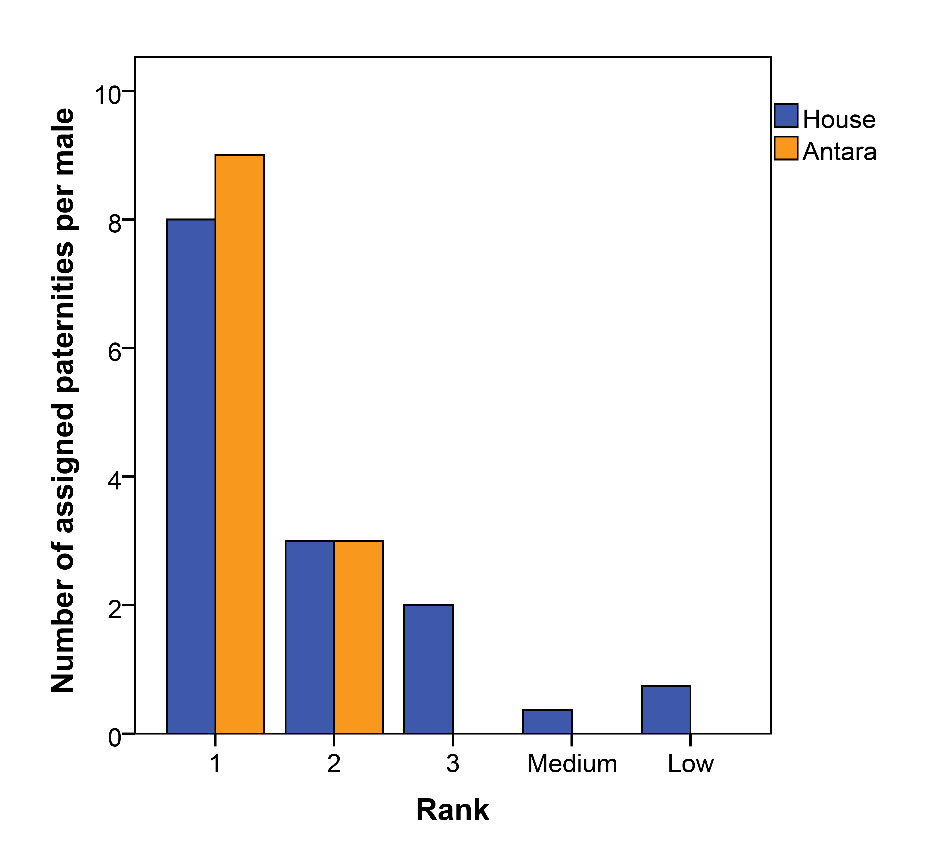


**Figure S1:** Number of offspring sired per male rank: 100% of assigned offspring was sired by the top two ranking males in Antara group and 81% by the top three in House group. Note that there are on average 2.7 males in both the medium and the low rank category.

High-ranking males (ranks 1-2) maintained a high rank for longer if they were in the same group as a related male (LMM: N=6 without related males, N=7 with related males, χ^2^_ML_: *P* = 0.041 (Table S6). See main text for results including rank 1-3). Related males were present on average for 72% (range 17% - 100%, N=7) of a male’s tenure (Fig S2).

|  | **β** | **S.E.** | **t-value** | **p-value** |
| --- | --- | --- | --- | --- |
| Intercept | 13.83 | 5.49 | 2.52 |  |
| Predictor variable  (Relative Yes/No) | 16.60 | 7.48 | 2.22 | 0.041 |

Table S6: In the presence of a related male the two top-ranking males can maintain their rank significantly longer compared to males without a related male: χ^2^_ML_ = 4.18.


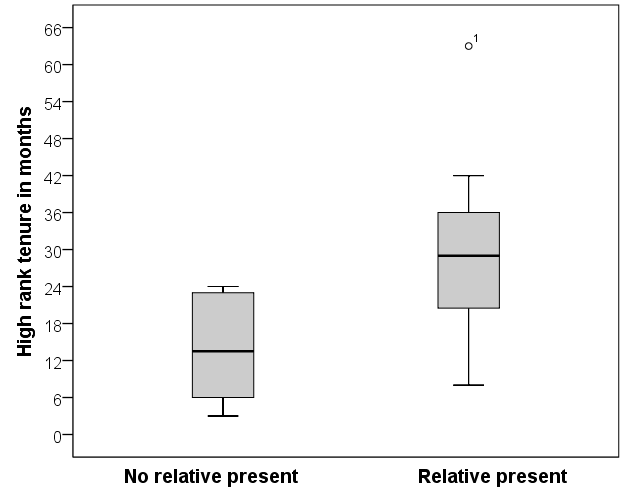
**Figure S2:** Effects of related males present in a group on high-rank tenure. High-ranking males (rank 1 and 2) with related males in the same group maintain a high rank for longer compared to males without co-residing related males.

**Results from Approach Two (A2) to assign males to the ‘related’ or ‘unrelated’ category**

We used two different approaches to assign males to the ‘related’ or ‘unrelated’ category. The first approach (A1) is based on the range of observed pairwise genetic relatedness values of empirically determined half-siblings and is presented in the main document. In the second approach (A2) we utilized the distribution of 1000 simulated r-values of unrelated and half-siblings dyads each from the Coancestry analysis (see main text for further details). We carried out all statistical analyses twice: once with the males categorised as ‘related’ or ‘unrelated’ according to A1 and once according to A2. The results are highly consistent. Thus, we report the results from A1 in the main text and the ones from A2 below.

**Comparison of residence time and high rank tenure of related and unrelated males**

Males with a related co-residing partner in a group (22; 13 censored) had a significantly higher probability to remain in the group compared to males without related partners (9; 4 censored) (MECM, χ^2^_ML_: *P* = 0.006, Table S7). Neither the type of dispersal (i.e. natal or non-natal), nor the interaction between the presence of relatives and dispersal type had a significant effect on residence time (MECM, χ^2^_ML Mode of Dispersal_: *P* = 0.62, χ^2^_ML Interaction_: *P* = 0.97, Table S7).

|  | **β** | **S.E.** | **z-value** | **p-value** |
| --- | --- | --- | --- | --- |
| Presence of relatives (yes/no) | -3.969 | 1.51 | -2.63 | 0.009 |
| Type of dispersal (natal/non-natal) | 1.244 | 2.48 | 0.50 | 0.62 |
| Interaction between presence of relatives and type of dispersal | 0.010 | 2.63 | 0.04 | 0.97 |

Table S7: Results from the Mixed Effects Cox model of A2

Due to the smaller dataset in A2, the GLMM did not converge and thus results need to be interpreted with caution, especially since the standard errors are large (GLMM , N = 9 without related males, 56% stayed for a year, N = 16 with related males, 100% stayed for a year, χ^2^_ML_: *P* = 0.007, Table S8).

|  | **β** | **S.E.** | **z-value** | **Pr (>\|z\|)** | **p-value** |
| --- | --- | --- | --- | --- | --- |
| Intercept | 10.35 | 23.21 | 0.45 | 0.66 |  |
| Relative at entry yes/no | 122.80 | 1.678e+7 | 0.000 | 1.00 | 0.007 |

Table S8: Relative at entry: χ2ML = 7.22

To still investigate whether the presence of a related male affects a male’s first year residence, we ran a Fisher’s exact test in R (Fay 2010) on a dataset that excluded multiple sightings of three individuals that had entered the same group on multiple occasions. One male showed a markedly different behaviour in that on one occasion he actually left the group within a year while a relative was present, while on the second occasion he stayed. We carried out two tests. In the first test we coded the male for having stayed while a relative was present (Fisher’s exact test, N=23, *P* = 0.032), while in the second test we coded the male as having emigrated before the end of his first year in the group (Fisher’s exact test, N=23, *P* = 0.083).

**R codes used for Mixed Effects Cox models and (General) Linear Mixed Effects models**

All Mixed Effects Cox models (MECM) were computed in R using coxme as described in Therneau (2015). The p-values are obtained from a maximum-likelihood (ML) estimate. In our case, we compared the following two models:

*efit1 <- coxph(Surv(TotalDur, AllData) ~ Relative*NatalDisperser, A1)*

*efit2 <- coxme(Surv(TotalDur, AllData) ~ Relative*NatalDisperser + (1|ID/Pop), A1)*

*efit1* corresponds to the model without random effects, which is compared to *efit2*, the model containing random effects. The variable “*TotalDur*” corresponds to the number of months a male has been observed as a member of one of the two study groups (House or Antara). “*AllData*” indicates whether we have a complete record of a male’s time of residence in a group or not, in terms of the survival analysis this is used to identify censored data. Since we are interested whether residence time in a group is influenced by the mode of dispersal (natal or non-natal) or the presence of relatives, we included these as fixed effects (they are encoded as “*NatalDisperser*” and “*Relative*” in the models). We included the presence of relatives and the type of dispersal as an interaction to investigate whether the presence of relatives has a different effect depending on whether a male is a natal or a non-natal disperser. Finally, our random effects consist of individual males (“*ID*”) and the study group, Houser or Antara (“*Pop*”). The dataset is the last term written in the equations and relates to either Approach 1 *“A1”* or Approach 2.

We used the R package *lme4* (Bates 2014) for the (General) Linear Mixed Effects models ((G)LMM). The GLMMs allowed us to assess whether related males (“*RelativeAtEntry*”) or peers provided some sort of entry support for new immigrants which made them stay in the group for a year (“*TwelveMonthsYN”*). As well as in the MECM we entered individuals (“*ID”*) nested within populations (“*Pop*”) as random effects, resulting in the following model and null model without the effect in question (presence of relatives or peers at entry):

*gm1 <- glmer(cbind(TwelveMonthsYN) ~ RelativeAtEntry + (1|Pop/ID), data=A1ZeroToTwelve, family=binomial)*

*gm2 <- glmer(cbind(TwelveMonthsYN) ~ 1 + (1|Pop/ID), data=A1ZeroToTwelve, family=binomial)*

The models were fitted by the Laplace approximation and compared in a maximum likelihood ratio test (ML) using the anova () function:

*anova (gm1,gm2)*

The Linear Mixed Effects models to assess whether the presence of “*Relatives*” has an effect on “*Tenure*” were entered in R as written below:

*tenure.model = lmer(Tenure ~ Relatives + (1|Pop/ID), data=A1Tenure)*

*tenure.null = lmer(Tenure ~ 1 + (1|Pop/ID), data=A1Tenure)*

We entered individual males (“ID”) nested within population (“Pop”) as random effects. The p-value was calculated in the same manner as for the GLMM by using the anova () function:

*Anova(tenure.nulll,tenure.model)*

**References:**

Bates D, Maechler M, Bolker BM, Walker S (2014) *lme4*: Linear mixed-effects models using Eigen and S4.

Brownstein MJ, Carpten JD, Smith JR (1996) Modulation of non-templated nucleotide addition by taq DNA polymerase: Primer modifications that facilitate genotyping. *Biotechniques* **20**, 1004-1006, 1008-1010.

Dakin EE, Avise JC (2004) Microsatellite null alleles in parentage analysis. *Heredity* **93**, 504-509.

Fay MP (2010) Two-sided Exact Tests and Matching Confidence Intervals for Discrete Data. *R Journal* **2**, 53-58.

Higashino A, Osada N, Suto Y*, et al.* (2009) Development of an integrative database with 499 novel microsatellite markers for *Macaca fascicularis*. *BMC Genetics* **10**, 24.

Lynch M (1988) Estimation of relatedness by DNA fingerprinting. *Molecular Biology and Evolution* **5**, 584-599.

Lynch M, Ritland K (1999) Estimation of pairwise relatedness with molecular markers. *Genetics* **152**, 1753-1766.

Marshall TC, Slate J, Kruuk LEB, Pemberton JM (1998) Statistical confidence for likelihood-based paternity inference in natural populations. *Molecular Ecology* **7**, 639-655.

Peakall R, Smouse PE (2006) GENALEX 6: genetic analysis in Excel. Population genetic software for teaching and research. *Molecular Ecology Notes* **6**, 288-295.

Queller DC, Goodnight KF (1989) Estimating relatedness using genetic-markers. *Evolution* **43**, 258-275.

Rice WR (1989) Analyzing tables of statistical tests. *Evolution* **43**, 223-225.

Ritland K (1996) Estimators for pairwise relatedness and individual inbreeding coefficients. *Genetics Research* **67**, 175-185.

Rousset F (2008) GENEPOP ' 007: a complete re-implementation of the GENEPOP software for Windows and Linux. *Molecular Ecology Resources* **8**, 103-106.

Therneau TM (2015) Mixed Effects Cox Model, Rochester, Minnesota.

Wang J (2007) Triadic IBD coefficients and applications to estimating pairwise relatedness. *Genetics Research* **89**, 135-153.

Wang JL (2002) An estimator for pairwise relatedness using molecular markers. *Genetics* **160**, 1203-1215.
